# Oral health in patients with end‐stage renal disease: A scoping review

**DOI:** 10.1002/cre2.479

**Published:** 2021-08-29

**Authors:** Alexa Laheij, Wietse Rooijers, Lela Bidar, Lema Haidari, Aegida Neradova, Ralph de Vries, Frederik Rozema

**Affiliations:** ^1^ Department of Oral Medicine, Academic Center for Dentistry Amsterdam University of Amsterdam and VU University Amsterdam Netherlands; ^2^ Department of Nephrology Dianet Amsterdam, and Amsterdam UMC Netherlands; ^3^ Medical Library VU University Amsterdam Netherlands; ^4^ Department of Oral and Maxillofacial Surgery Amsterdam UMC, location AMC Netherlands

**Keywords:** dental caries, end‐stage renal disease, periodontitis, xerostomia

## Abstract

**Objectives:**

In patients with end stage, renal disease a high rate of morbidity and mortality is present. Studies suggest that end stage renal disease may affect oral health. Therefore, the aim of this study was to perform a scoping review on periodontal disease, dental caries, xerostomia, and hyposalivation in end stage renal disease patients.

**Materials and methods:**

A literature search (in PubMed and Embase.com) was performed up to September 29, 2020, in collaboration with a medical information specialist. Included outcome variables were the community periodontal index, probing pocket depth, gingival index, bleeding on probing, decayed‐missing‐filled‐teeth, carious‐absent‐obturated index, Xerostomia Inventory and the (un)stimulated whole salivary flow rate.

**Results:**

Forty three out of 1293 studies were included in the final review comprising 7757 end stage renal disease patients. The average age was 58.3 ± 29.4 years. 28.2%–78.8% of patients reported xerostomia and the (un)stimulated salivary flow rates were significantly lower. Higher community periodontal index scores were measured in end stage renal disease patients. More decayed‐missing‐filled‐teeth were recorded, but no differences were found between groups.

**Conclusions:**

Xerostomia and hyposalivation were highly prevalent in end stage renal disease patients. Patients have more deepened pockets, but an equal number of carious teeth compared to healthy controls.

## 1 INTRODUCTION

The global prevalence of chronic kidney disease (CKD) including its most critical stage; end stage renal disease (ESRD), is estimated to be between 13.9% and 0.1%, respectively. Diabetes, hypertension and an older age are significant risk factors for developing CKD and ESRD (Hill et al., 2016). The disease is more common among women than men (Carrero, Hecking, Chesnaye, & Jager, 2018). The decline in kidney function causes waste products to accumulate inside the body (Brennan, Collett, Josland, & Brown, 2015; Webster, Nagler, Morton, & Masson, 2017) and causes symptoms like reduced mobility, lack of energy, reduced appetite, and sleeping disorders (Webster et al., 2017). Complications of CKD include fluid retention, anemia (Babitt & Lin, 2012; Bello et al., 2017), and it is an independent risk factor for cardiovascular disease and mortality (Matsushita et al., 2012). This risk rises with the progression of kidney dysfunction (Manjunath et al., 2003).

Individuals with a severe loss of kidney function (ESRD) may require renal replacement therapy when noninvasive measures no longer provide symptom relief (Glorieux & Tattersall, 2015). Currently, the best treatment option for renal replacement therapy is a kidney transplantation. Until a donor‐kidney becomes available dialysis therapy is necessary.

Besides systematic complications from CKD and ESRD, oral health may be negatively affected by the disease itself, its treatment and its associated lifestyle alterations. Estimates are that oral diseases are present in almost 90% of dialysis patients (De Rossi & Glick, 1996). A diminished oral health in ESRD patients was frequently reported (Ruospo et al., 2014). Dry mouth is often present and may be associated with a fluid‐restricted diet and hemodialysis drug therapy (Proctor, Kumar, Stein, Moles, & Porter, 2005). In the long‐term, patients with a dry mouth are predisposed to develop more caries, periodontal disease, and mucosal lesions (Bossola & Tazza, 2012; Porter, Scully, & Hegarty, 2004). Moreover, oral symptoms, especially a lower salivary flow and a lower number of teeth, are related to a lower oral health related quality of life (Ruokonen et al., 2019). Therefore, keeping good oral health is of key importance for ESRD patients.

Recently, several studies concerning the oral health in ESRD patients were performed. Therefore, the aim of this scoping review is to update the available literature on periodontal disease, dental caries, xerostomia, and hyposalivation in ESRD patients.

## 2 METHODS

### 2.1 Search strategy

A literature search was performed based on the Preferred Reporting Items for Systematic Reviews and Meta‐Analyses (PRISMA) statement (Moher, Liberati, Tetzlaff, & Altman, 2009). Additionally, the Preferred Reporting Items for Systematic Reviews and Meta‐analyses extension for Scoping Reviews (PRISMA‐ScR) (Tricco et al., 2018) was used.

To identify all relevant publications, a systematic search in the bibliographic databases PubMed and Embase.com was conducted from inception to September 29, 2020, in collaboration with a medical information specialist. The following terms were used (including synonyms and closely related words) as index terms or free‐text words: “Chronic renal insufficiency”, “Kidney failure”, “Renal dialysis”, “Hemodialysis”, “Periodontitis”, “Xerostomia”.

The references of the identified articles were searched for relevant publications. Duplicate articles were excluded. All languages were accepted. The full search strategies for all databases can be found in Supplementary Table S1.

### 2.2 Selection process

Three reviewers (AL, LB and LH) independently screened the potentially relevant titles and abstracts for eligibility using the review manager Rayyan QCRI (Ouzzani, Hammady, Fedorowicz, & Elmagarmid, 2016). If necessary, the full text article was checked for the eligibility criteria. Differences in judgment were resolved through a consensus procedure. Studies were included if they met the following criteria: (a) Adult patients ≥18 years old with chronic kidney disease stage G5 (eGFR <15 ml/min/1.73 m^2^ body surface area) with or without dialysis therapy (including patients waiting for a transplant); (b) studies on oral health including any of the following: periodontal disease, dental caries, xerostomia, or hyposalivation; (c) studies assessing the influence of renal insufficiency on oral health; (d) observational studies (cohort, case–control and cross‐sectional studies); (e) written in English, Dutch or translated. We excluded studies if they were: (a) studies in which the type or severity of renal insufficiency was not specified; (b) studies in which patients were suffering from acute kidney injury or acute‐on‐chronic renal failure or in which patients were examined after receiving a renal transplant; (c) studies assessing the influence of oral health on renal insufficiency; (d) letters or comments on articles, study protocols, preliminary studies, pilot studies, case series (<4 patients) or case reports.

### 2.3 Data assessment

The full text of the selected articles was obtained for further review. Three reviewers (AL, LB and LH) independently evaluated the methodological quality of the full text papers using the Joanna Briggs Institute Critical Appraisal Checklist for Studies Reporting Prevalence Data (Munn, Sandeep, Lisy, Riitano, & Tufanaru, 2015), Supplementary Table S2. It consists of nine questions regarding the possibility of bias at the study and outcome level. The checklist was mainly used to assess the overall body of evidence and validity of the results.

### 2.4 Data extraction

The community periodontal index of treatment needs (CPI[TN]), probing pocket depth (PPD), the gingival index (GI) (Löe & Silness, 1963), bleeding on probing (BOP), and PISA scores were extracted to assess the periodontal condition; the decayed‐missing‐filled‐teeth (DMFT) and carious‐absent‐obturated (CAO) indexes for the caries history, the xerostomia inventory (XI) (Thomson, Chalmers, Spencer, & Williams, 1999), or any survey/visual analogue scale (VAS) reporting dry mouth for xerostomia and the (un)stimulated whole salivary flow rate (UWSFR/SWSFR) for hyposalivation.

The relevant values of the periodontal variables (in view of periodontitis) were probing pocket depths >3 mm, corresponding with CPI scores of 3 (pocket depth 4–5 mm) and 4 (pocket depth ≥ 6 mm) and moderate to severe inflammation, indicated by GI scores of 2 (moderate) and 3 (severe) and sites with (immediate) bleeding on probing.

Results from individual studies were represented in tables. Means and SDs that were available for subgroups were recalculated for the whole group if applicable.

## 3 RESULTS

### 3.1 Search results

**Figure 1:**
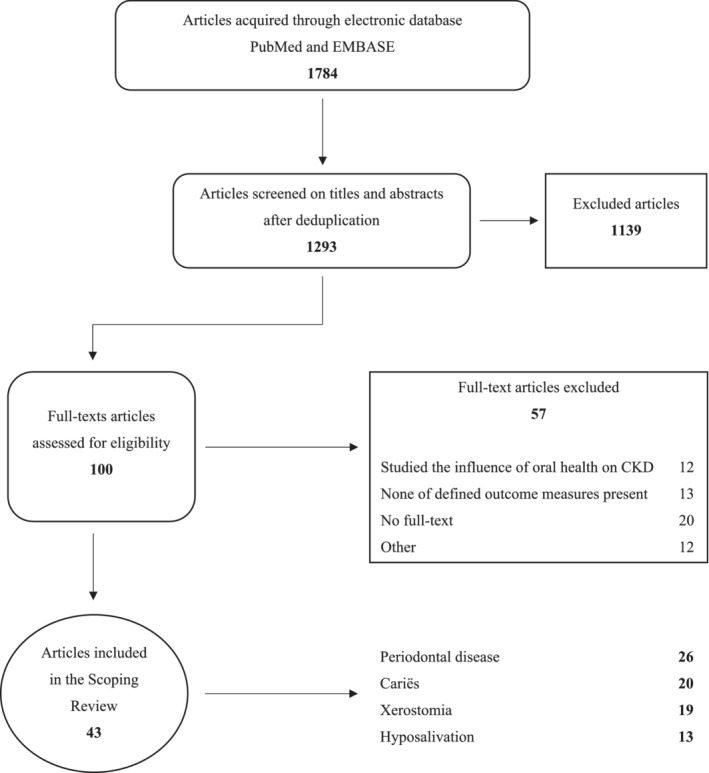
Flow diagram of the study selection process

The literature search generated a total of 1784 references: 722 in PubMed and 1062 in Embase.com. After removing duplicates of references that were selected from more than one database, 1293 references remained. In total, 43 articles were included for data extraction (Table 1): 1 RCT, 36 cross‐sectional studies, three case–control studies, two cohort studies and one longitudinal study (Al‐Wahadni & Al‐Omari, 2003; Bayraktar et al., 2008; Bots et al., 2004; Bots et al., 2006; Bots et al., 2007; Bruzda‐Zwiech, Szczepańska, & Zwiech, 2014; Bruzda‐Zwiech, Szczepańska, & Zwiech, 2018; Chuang, Sung, Kuo, Huang, & Lee, 2005; de la Rosa García, Mondragón Padilla, Aranda Romo, & Bustamante Ramírez, 2006; Dirschnabel et al., 2011; Eltas, Tozoğlu, Keleş, & Canakci, 2012; Gautam et al., 2014; Gavaldá et al., 1999; Honarmand, Farhad‐Mollashahi, Nakhaee, & Sargolzaie, 2017; Jain et al., 2014; Jung & Chang, 2020; Kaushik et al., 2013; Kho, Lee, Chung, & Kim, 1999; Križan Smojver, Altabas, Knotek, Bašić Jukić, & Aurer, 2020; López‐Pintor, López‐Pintor, Casañas, de Arriba, & Hernández, 2017; Malekmakan, Haghpanah, Pakfetrat, Ebrahimic, & Hasanlic, 2011; Marakoglu, Gursoy, Demirer, & Sezer, 2003; Marinoski, Bokor‐Bratic, Mitic, & Cankovic, 2019; Menezes et al., 2019; Misaki, Fukunaga, & Nakano, 2020; Murali, Narasimhan, Periasamy, & Harikrishnan, 2012; Naruishi et al., 2016; Oliveira et al., 2020; Pallos et al., 2020; Palmer et al., 2016; Parente et al., 2018; Perozini et al., 2017; Schmalz et al., 2016; Schmalz et al., 2017; Schütz et al., 2020; Sekiguchi, Pannuti, Silva, Medina‐Pestana, & Romito, 2012; Sobrado Marinho et al., 2007; Swapna et al., 2013; Tadakamadla, Kumar, & Mamatha, 2014; Torres et al., 2010; Yue et al., 2018; Zhao et al., 2014). The flow chart of the search and selection process is presented in Figure 1.

**Table 1:** Characteristics of the studies included

First author	Year	Design	Target population	*N* and gender	Healthy controls	Age ± standard deviation	Mean dialysis time (months)	ESRD causes	Outcomes	Measurement
Jung	2020	RCT	HD	53: 30M/23F	No	64.4 ± 1.3	66.7 ± 56.2		Xerostomia, hyposalivation	VAS, UWSFR
Krizan Smojver	2020	Cross sectional	HD and PD	80: 47M/42F	No	59.4	27.6		Periodontitis	PISA
Misaki	2020	Prospective cohort	HD	89:48M/32F	No	67.3 ± 12.2	91.2 ± 70.8	glomerulonephritis (28.8%), diabetic nephropathy (26.3%), nephrosclerosis (31.3%), other (13.6%)	Periodontitis, caries	DMFT, PPD
Oliveira	2020	Cross sectional	HD	180:99M/81F	No	52.0 ± 14.3	<12 months: 23.3%; 12–36 months: 33.9%; >36 months: 42.8%		Periodontitis, dental caries, xerostomia	PI, BOP, Xerostomia, untreated dental caries
Pallos	2020	Cross sectional	HD	40	No				Periodontitis	CAL, PPD, PI, GI
Schütz	2020	Cross sectional	ESRD not on dialysis	32:19 M/13F	No	20–49 years: 18.7%; 50–64 years: 34.4%; ≥65 years: 46.9%	<5 years: 75%; ≥5 years: 25%		Periodontitis,	CAL, PPD, BOP, PI
Marinoski	2019	Cross sectional	HD	25: 18M/7F	Yes	54.9 ± 13.6			Xerostomia, hyposalivation	Xerostomia, UWSFR
Menezes	2019	Case–control	HD	107: 59M/48F	Yes	44.6			Caries	DMFT
Viana‐Rojas	2019	Cross sectional	HD	111: 57M/54F	No	42.9 ± 17.8	27.1 ± 30.5		Periodontitis	GI, PPD, BOP
Bruzda‐Zwiech	2018	Cross sectional	HD with or without DM	97: 83M/42F	No	58.3 ± 12.2	12.7 ± 6.9		Xerostomia, hyposalivation	XI, UWSFR
Parente	2018	Cross sectional	HD	75: 43M/32F	Yes	44.9 ± 19.1			Periodontitis	BOP
Yue	2018	Cross sectional	HD	30: 15M/15F	Yes	48.5 ± 12.7	68.8 ± 46.7		Caries	DMFT
Schmalz	2017	Cross sectional	HD with or without DM	159: 102M/57F	No	68.3 ± 12.2	47.3 ± 44.1		Periodontitis, caries, hyposalivation	PPD, BOP, DMFT, USWSFR, SWSFR
Perozini	2017	Cross sectional	HD	28: 16M/12F	No	49.4 ± 11.9			Periodontitis	PPD, GI
López‐Pintor	2017	Cross sectional	HD	50: 35M/15F	No	66.6 ± 14.0	46.0 ± 44.9	DM 32%, Hypertension 16%	Xerostomia, hyposalivation	Xerostomia, Xerostomia VAS, UWSFR (*n* = 30), SWSFR (*n* = 30)
Honarmand	2017	Cross sectional	HD	30: 21M/9F	Yes	38.2 ± 16.9			Xerostomia	Dry mouth
Palmer	2016	Prospective cohort	HD	4205: 2426M/1779F	No	61.6 ± 15.6	77.5 ± 59.1		Periodontitis, caries, xerostomia, hyposalivation	CPI (scores not reported), PPD, BOP, DMFT, SWSFR, Dry mouth
Schmalz	2016	Cross sectional	HD	35: 21M/14F	No	56.4 ± 11.1	66.0 ± 76.8		Caries	DMFT
Naruishi	2016	Cross sectional	HD with or without DM	119: 79M/40F	No	61.0 ± 10.5			periodontitis	CPI
Bruzda‐Zwiech	2014	Cross sectional	HD	111: 64M/47F	No	59.1 ± 13.6	14.7 ± 8.9	DM 36%, Hypertension 14%	Xerostomia, hyposalivation	XI, UWSFR
Zhao	2014	Case control	HD	102: 59M/43F	Yes	58.4 ± 14.1	time on dialysis in yrs (1–2 / 2–5 / 5>)	DM 27%, Hypertension 15%	Periodontitis	CPI
Gautam	2014	Cross sectional	Dialysis	206: 167M/39F	No	46.8 ± 12.8	time on dialysis in years (<1/1–3/<3)		Oeriodontitis	CPI
Tadakamadla	2014	Cross sectional	Chronic Kidney Disease	19	Yes				Periodontitis, caries	CPI, GI, DMFT
Jain	2014	Cross sectional	HD	400: 268M/132F	Yes	51.3 ± 16.1	time on dialysis (0–3/ 4–6/7–9/10–12/<12)		Periodontitis, caries	CPI, DMFT
Swapna	2013	Cross sectional	HD with or without DM	97: 62M/35F	No	54.6 ± 11.3	42.9 ± 21.0		Periodontitis, caries, xerostomia	CPI, DMFT, dry mouth
Kaushik	2013	Cross sectional	HD	100: 61M/39F	Yes	44.4 ± 7.5	26.3 ± 11.5		Xerostomia, hyposalivation	Dry mouth, UWSFR (*n* = 25), SWSFR (*n* = 25)
Murali	2012	Cross sectional	HD with or without DM	100: 62M/38F	No	(25–79)			Periodontitis, caries, xerostomia	CPI, DMFT, Xerostomia
Eltas	2012	Cross sectional	PD with or without DM	49: 21M/28F	No	42.2	38.6 ± 6.7		Periodontitis, caries, xerostomia, hyposalivation	CPI, DMFT, XI (scores not reported), UWSFR
Sekiguchi	2012	Cross sectional	HD	94: 51M/43F	No		time on dialysis in years (<3/<3)		Periodontitis, caries	PPD, GI, BOP, DMFT
Malekmakan	2011	Cross sectional	HD	72: 48M/24F	No	53.4 ± 15.3	36.9 ± 33.8		Caries, xerostomia	DMFT, dry mouth
Dirschnabel	2011	Cross sectional	HD	46: 23M/23F	Yes	48.0 ± 14.0	38.1		Xerostomia	Xerostomia
Torres	2010	Cross sectional	HD	16: 12M/4F	No	41.7 ± 7.2	29.1 ± 22.4		Periodontitis	PPD, GI
Bayraktar	2008	Cross sectional	HD and PD	116: 55M/61F	Yes	44.7 ± 13.2	36.4 ± 23.9	DM 12%, Hypertension 20%	Periodontitis	PPD, GI
Sobrado Marinho	2007	Case control	HD	28	Yes				Caries	DMFT
Bots	2007	longitudinal OS	Dialysis	23	No				Periodontitis, caries, xerostomia, hyposalivation	PPD, BOP, DMFT, DMFS, XI, UWSFR, SWSFR
De la Rosa García	2006	Cross sectional	Dialysis with DM	99: 37M/62F	No	57.9 ± 11.6			Xerostomia	Xerostomia
Bots	2006	Cross sectional	Dialysis	42: 30M/12F	Yes	42.6 ± 9.2	28.6 ± 16.9		Periodontitis, caries	PPD, BOP, DMFT, DMFS
Chuang	2005	Cross sectional	HD with or without DM	128: 58M/70F	No	58.8 ± 11.8	40.6 ± 33.3		Periodontitis, caries, xerostomia	CPI, DMFT, Xerostomia VAS
Bots	2004	Cross sectional	HD	94: 64M/30F	No	56.3 ± 16.6	35.8 ± 31.0	DM 5%, Hypertension 16%	Xerostomia, hyposalivation	XI, UWSFR, SWSFR
Marakoglu	2003	Cross sectional	HD	36: 20M/16F	Yes	50.4 ± 14.2			Periodontitis	PPD, GI
Al‐Wahadni	2003	Cross sectional	Dialysis	47: 24M/23F	No	42.9 ± 12.5	time on dialysis in years (<1/1–3/<3)		Periodontitis, caries	PPD, GI, DMFT
Gavaldá	1999	Cross sectional	HD	105: 53M/52F	Yes	58.9 ± 14.9	59.8 ± 43.9	DM 7%, Hypertension 10%	Caries, hyposalivation	CAO, UWSFR, SWSFR
Kho	1999	Cross sectional	HD	82: 54M/28F	Yes	33.5 ± 10.3	22.0		Xerostomia, hyposalivation	Dry mouth, UWSFR (*n* = 22)

Abbreviations: BOP, bleeding on probing; CAO, carious‐absent‐obturated; CPI, community periodontal index; DM, diabetes mellitus; DMFT, decayed‐missing‐filled teeth; ESRD, end‐stage renal disease; HD, hemodialysis; OS, observational studies; PD, peritoneal dialysis; PPD, probing pocket depth; SWSFR, stimulated whole salivary flow rate; UWSFR, unstimulated whole salivary flow rate; XI, xerostomia inventory.

### 3.2 Study characteristics

In total, 7757 patients were included, 4558 (58.8%) were male, 3233 (41.2%) female. The mean age was 58.3 ± 29.4 years (*n* = 7335) and the mean dialysis time of the subjects was 67.8 ± 73.5 months (*n* = 5812). One study selected ESRD patients but did not mention whether they were on dialysis, and one selected ESRD patients that were not on dialysis. Thirty studies focused on hemodialysis, one focused on peritoneal dialysis, one compared patients on hemodialysis and peritoneal dialysis, and six studied ESRD patients, who were on either hemodialysis or peritoneal dialysis. Of these studies, seven considered whether the length of time spent on dialysis had any consequences on oral health and eight studies studied diabetic dialysis patients. Fifteen studies compared their subjects with a healthy control group, of which 12 studies used age‐ and/or gender‐matched controls.

### 3.3 Quality assessment

The results of the critical appraisal can be found under Supplementary Table S2. No study did random probabilistic sampling in a pool of ESRD subjects. Loss to follow up (question 9) was only applicable to the longitudinal study. All studies scored well on the critical appraisal. The majority of the studies had a cross‐sectional design.

### 3.4 Periodontal disease

Studies reported different indices related to periodontal health. The prevalence of CPI 3 in ESRD patients varied between 7.5% and 57.0% and of CPI 4 between 1.0% and 78.9% (Table 2). All subjects (both ESRD patients and healthy controls) showed signs of gingival inflammation. Based on the mean values, ESRD patients had no to mild gingival inflammation (GI 0–1) (Bayraktar et al., 2008; Sekiguchi et al., 2012; Torres et al., 2010), mild to moderate inflammation (GI 1–2) (Marakoglu et al., 2003; Pallos et al., 2020; Perozini et al., 2017) and moderate to severe inflammation (GI 2–3) (Tadakamadla et al., 2014). One study reported higher GI in ESRD patients compared to control (Tadakamadla et al., 2014; Viana‐Rojas et al., 2019). No association was found between the duration of dialysis history and higher GI scores (Al‐Wahadni & Al‐Omari, 2003; Sekiguchi et al., 2012).

**Table 2:** Periodontal parameters

	ESRD	Control	*p* value	ESRD	Control	*p* value
CPI	CPI 3 (%)			CPI 4 (%)		
Naruishi 2016	Not specified			Not specified		
Gautam 2014	44.3			39.3		
Jain 2014	7.5	10.0	–			
Tadakamadla 2014	15.8	19.3	–	78.9	0.0	–
Zhao 2014	41.2	23.2	<0.001	38.7	9.7	<0.001
Swapna 2013	57.0			12.6		
Murali 2012	26.0			0.0		
Chuang 2005	43.8			21.1		

PPD	Mean (standard deviation)			% >3 mm or >3.5 mm		
Misaki 2020				86.3		
Pallos 2020	2.9 (0.9)					
Schütz 2020	2.7 (0.6)					
Vaiana‐Rojas 2019	3.0 (1.0)					
Perozini 2017	2.8 (1.0)					
Schmalz 2017	3.4 (1.1)					
Palmer 2016	1.0					
Sekiguchi 2012	1.8 (0.4)					
Torres 2010	1.8 (0.3)					
Bayraktar 2008	1.9 (0.5)	1.9 (0.6)	NS			
Bots 2007				5.7		
Bots 2006				4.3	13.1	NS
Al‐Wahadni 2003	2.9 (0.6)			29.8		
Marakoglu 2003	1.8 (0.6)	1.8 (0.8)	NS			

GI	Mean (standard deviation)			≥2 (%)		
Vaiana‐Rojas 2019	2.2 (0.4)					
Perozini 2017	1.6 (0.4)					
Tadakamadla 2014	2.4 (0.4)	0.9 (0.4)	<0.05			
Sekiguchi 2012	0.9 (0.4)					
Torres 2010	0.6 (0.4)					
Bayraktar 2008	0.3 (0.4)	0.2 (0.3)	NS			
Al‐Wahadni 2003				55		
Marakoglu 2003	1.5 (0.3)	1.4 (0.5)	NS			

BOP	Mean % (standard deviation)					
Schütz 2020	63.9 (30.4)					
Vaiana‐Rojas 2019	36.7 (60.5)					
Parente 2018	15.3 (23.2)					
Schmalz 2017	10 (12.3)					
Palmer 2016	9.4					
Sekiguchi 2012	29.0 (15.0)					
Bots 2007	29.5 (25.4)					
Bots 2006	37.9	34.0	NS			

PISA	Mean (standard deviation)					
Krizan Smojver 2020	644.7 (510.7)					

No study found significantly deeper pockets in ESRD patients compared to healthy controls (Table 4). Of the three studies considering the dialysis history (Al‐Wahadni & Al‐Omari, 2003; Bots et al., 2006; Sekiguchi et al., 2012), only Sekiguchi et al. found a significant correlation between time on dialysis and higher pocket depths (*r* = 0.391; *p* < 0.001). This is in contrast with results from Bots et al., who did not observe an increase in pocket depth in dialysis patients after a 2‐year follow‐up (Bots et al., 2007).

The percentages BOP in ESRD patients ranged from 9.4% (low) to 63.9% (high). There was no significant difference in BOP between dialysis patients and healthy controls (Bots et al., 2006). The time spent on dialysis was not associated with higher BOP levels (Palmer et al., 2016; Sekiguchi et al., 2012). Bots et al. found a significant decrease in BOP levels of dialysis patients after 2 years (Bots et al., 2007). Schütz et al. described that 59.4% of ESRD patients was diagnosed with severe periodontitis (Schütz et al., 2020). One study reported the total surface area of inflamed periodontal tissue (PISA score) per patient (Križan Smojver et al., 2020).

### 3.5 Caries

There was a huge spread in mean DMFT scores ranging from 1.4 (almost no carious teeth) to 26.0 (almost all teeth were carious) (Table 3). Two studies found higher DMFT scores in patients who were longer on dialysis (Al‐Wahadni & Al‐Omari, 2003) (Sekiguchi et al., 2012), while three studies did not (Bots et al., 2006; Chuang et al., 2005; Jain et al., 2014). The study of Gavaldá et al. used the Carious, Absent and Obturated (CAO) Index, according to the World Health Organization (WHO) guidelines of 1987 (Gavaldá et al., 1999). Oliveira et al. reported that 82.7% of patients on hemodialysis had untreated dental caries (Oliveira et al., 2020). No significant differences were found when comparing the DMFT scores of hemodialysis patients to those of healthy controls.

**Table 3:** Mean DMFT score

DMFT	ESRD	Control	*p* value
Misaki 2020	18.9 (7.0)		
Menezes 2019	14.8 (8.0)	16.4 (7.7)	–
Yue 2018	4.4 (3.9)	2.3 (2.5)	<0.01
Schmalz 2017	20.0 (5.7)		
Palmer 2016	22.0		
Schmalz 2016	19.5 (5.8)		
Jain 2014	3.6	3.6	NS
Tadakamadla 2014	1.4 (1.5)	2.2 (1.8)	NS
Swapna 2013	12.3 (8.4)		
Eltas 2012	24.0		
Murali 2012	4.46 (4.98)		
Sekiguchi 2012	13.2 (5.2)		
Malekmakan 2011	18.6 (9.9)		
Sobrado Marinho 2007	14.4 (7.9)	15.2 (7.1)	–
Bots 2007	13.6 (8.5)		
Bots 2006	13.3 (7.5)	14.7 (6.4)	NS
Chuang 2005	16.2 (10.2)		
Al‐Wahadni 2003	8.5 (2.9)		

### 3.6 Dry mouth

Xerostomia prevalence ranged from 28.2% to 74.2% (Table 4). Xerostomia was reported significantly more often in diabetic peritoneal dialysis patients compared to nondiabetic peritoneal dialysis patients (Eltas et al., 2012), but no difference was found between diabetic hemodialysis patients and nondiabetic hemodialysis patients (Murali et al., 2012; Swapna et al., 2013). The incidence of dry mouth in the peritoneal dialysis patients increased as glycemic control worsened (<6% HbA1c: 37.5%; >6% HbA1c: 71.4%; *p* = 0.005) (Eltas et al., 2012). López‐Pintor et al. did not find an association between the number of prescribed drugs and an increased incidence of dry mouth symptoms (López‐Pintor et al., 2017).

**Table 4:** Xerostomia

	ESRD	Control	*p* value
Xerostomia (%)			
Oliveira 2020	35.0		
Marinoski 2019	60.0	0.0	–
Honarmand 2017	46.7	13.3	0.005
López‐Pintor 2017	56.0		
Palmer 2016	44.7		
Kaushik 2013	40.0		
Swapna 2013	70.1		
Eltas 2012	30.0		
Dirschnabel 2011	28.2	0.0	<0.05
Malekmakan 2011	48.6		
De la Rosa García 2006	44.4		
Bots 2004	74.2		
Kho 1999	32.9		

Xerostomia Inventory	Mean (standard deviation)		
Bruzda‐Zwiech 2018	34.1 (14.0)		
Bruzda‐Zwiech 2014	33.1 (10.7)		
Bots 2007	29.5 (7.5)		
Bots 2004	28.3 (9.1)		

VAS score	Mean (standard deviation)		
Jung 2020	5.8 (2.4)		
López‐Pintor 2017	4.0 (3.2)		
Chuang 2005	4.5 (1.8)		

The scores on the Xerostomia Inventory ranged from 28.3 to 34.1, indicating moderate to moderate high levels of xerostomia (Table 4). No association was found between the duration of dialysis and XI scores (Bots et al., 2004; Bots et al., 2007). Chuang et al. reported higher levels of xerostomia (VAS‐scores) in diabetic hemodialysis patients compared to nondiabetic hemodialysis patients. The VAS scores increased as glycemic control decreased (< 6% HbA1c: 3.6) to poor control (> 9% HbA1c: 5.9; *p* = 0.01) (Chuang et al., 2005).

The mean unstimulated whole salivary flow rate varied between 0.16 and 1.30 ml/min (Table 5). Two studies found a significantly lower UWSFR in hemodialysis patients compared to healthy controls (Kaushik et al., 2013; Kho et al., 1999). The prevalence of hyposalivation varied between 16.0% and 53.3%. The mean stimulated whole salivary flow rate varied between 0.42 and 3.80 ml/min (Table 5). Two studies described a lower SWSFR in hemodialysis patients compared to healthy controls (Gavaldá et al., 1999; Kaushik et al., 2013).

**Table 5:** UWSFR and SWSFR values in ml/min

	Unstimulated whole salivary flow rate: mean (standard deviation)			Stimulated whole salivary flow rate: mean (standard deviation)		
	ESDR	Control	*p* value	ESDR	Control	*p* value
Jung 2020	0.48 (0.49)					
Marinoski 2019	0.30 (0.16)	0.51 (0.19)	<0.001			
Bruzda‐Zwiech 2018	0.37 (0.31)					
López‐Pintor 2017	0.16 (0.17)			1.12 (0.64)		
Schmalz 2017	0.19 (0.22)			0.47 (0.45)		
Palmer 2016				0.83		
Bruzda‐Zwiech 2014	0.31 (0.28)					
Kaushik 2013	0.31 (0.01)	0.52 (0.06)	<0.001	0.66 (0.02)	1.16 (0.11)	<0.001
Eltas 2012	0.28 (0.08)					
Bots 2007	0.31 (0.19)			1.18 (0.80)		
Bots 2004	0.30 (0.22)			1.05 (0.70)		
Gavaldá 1999	1.30 (1.40)	1.40 (0.80)	NS	3.80 (1.9)	6.30 (3.8)	<0.001
Kho 1999	0.30 (0.18)	0.45 (0.25)	<0.05			

Hyposalivation (%)	Unstimulated saliva			Stimulated saliva		
Marinoski 2019	16.0	0.0	–			
Bruzda‐Zwiech 2018	33.0					
López‐Pintor 2017	53.3			36.7		
Bruzda‐Zwiech 2014	28.8					
Bots 2004	36.2					

## 4 DISCUSSION

Since the burden of oral symptoms in end stage renal disease patients may be high, the aim of this scoping review was to summarize the available literature on periodontal disease, dental caries, xerostomia, and hyposalivation in this patient group. Xerostomia and hyposalivation were highly prevalent in ESRD patients. Also, caries and periodontal disease were present. ERSD patients have more deepened pockets, but equal numbers of carious teeth compared to control patients. Only 38% of the included studies compared ESRD patients to a control group.

Dry mouth (xerostomia and hyposalivation) is highly prevalent in ESRD patients, and even though it is also present in the adult population (Jamieson & Thomson, 2020), it is more prevalent in ESRD patients compared to healthy controls. Dry mouth in ESRD patients may be caused by a fluid restricted diet, (multiple) medication use with dry mouth as side effect, the dialysis procedure itself, and/or salivary gland fibrosis and atrophy (Bossola & Tazza, 2012). Lack of saliva and dry mouth feeling may have several consequences for patients. They may lead to difficulty chewing, speaking and swallowing, taste alterations, halitosis, increased risk of oral infections, such as candidiasis, increased risk of (rapidly progressing) caries and periodontal disease, increased risk of fluid intake and interdialytic weight gain, and reduced quality of life (Bossola, 2019; de la Rosa García et al., 2006; Weisbord et al., 2005).

Diabetes mellitus was reported as a contributing factor for a dry mouth, patients with poor glycemic control experienced more oral dryness than patients with good glycemic control. High blood sugar levels lead to the excretion of large amounts of urine, which in turn leads to a decrease in intravascular fluid and hence an increase in oral dryness (Silveira Lessa et al., 2015). We found somewhat conflicting results when comparing diabetic and nondiabetic dialysis patients. Most studies reported more xerostomia (Bruzda‐Zwiech et al., 2018; Chuang et al., 2005; Eltas et al., 2012) and hyposalivation (Bruzda‐Zwiech et al., 2018; Eltas et al., 2012) in diabetic ESRD patients, while others did not find differences between diabetic and nondiabetic ESRD patients (Bruzda‐Zwiech et al., 2018; Murali et al., 2012; Schmalz et al., 2017; Swapna, Koppolu, & Prince, 2017).

As a complication of ESRD, patients have micro‐, and macrovascular complications (Bello et al., 2017; Ooi et al., 2011) and a weakened immune system, caused by immune cell dysfunction (monocytes, macrophages, B‐ and T‐lymfocytes) (Chonchol, 2006; Heinzelmann et al., 1999; Ismail et al., 2013). These complications can lead to a lowered immune reponse and a higher level of systemic inflammation (Bronze‐Da‐Rocha & Santos‐Silva, 2018; Heinzelmann et al., 1999; Salimi et al., 2014). Subsequently, a lowered immune response may result in an altered reaction to periodontal Gram‐negative pathogens (Ismail et al., 2013), potentially enabling these bacteria to take over the subgingival microbiome and induce periodontal breakdown.

As expected, periodontal inflammation was present in ESRD patients. Studies used different indices to estimate periodontal inflammation. In none of the studies the presence or absence of periodontitis could be determined. Compared to a healthy population, the periodontal status of ESRD patients was worse when scored with the CPI index. ESRD patients had more often pockets of 4‐5 mm, and ≥6 mm. However, results regarding the percentage of bleeding on probing, average pocket depth, or gingival inflammation were not as consistent. Only a couple of studies measured these parameters, and they consisted of low numbers of included patients (Marakoglu et al., 2003; Tadakamadla et al., 2014). One study reported more gingival inflammation in ESRD patients (Tadakamadla et al., 2014).

Diabetes mellitus is also associated with microvascular and macrovascular complications and is considered to be a risk factor for the development, progression, and severity of periodontitis (Verhulst, Loos, Gerdes, & Teeuw, 2019). Some studies compared diabetic versus nondiabetec ESRD patients. However, there were no significant differences between diabetic and nondiabetic ESRD patients regarding several periodontal parameters (Chuang et al., 2005; Murali et al., 2012; Naruishi et al., 2016; Swapna et al., 2013).

The total caries experience was mostly measured by the DMFT index. The variation in DMFT scores between studies was quite high. However, mostly high DMFT scores were measured in both ESRD patients and healthy subjects. As the DMTF index can only get higher when age increases, the high scores may partly be explained by the higher age of the included patients. Results comparing ESRD patients and healthy controls were conflicting and no clear difference between the groups was visible. However, in dialysis patients there was some evidence for a higher caries prevalence in patients with concomitant diabetes mellitus (Chuang et al., 2005; Eltas et al., 2012; Swapna et al., 2013).

Besides xerostomia, hyposalivation, caries and periodontitis, and other oral complications or oral symptoms could be present in ESRD patients. For instance, edentulousness, mucosal disease, bad oral hygiene, mucosal sensitivity, oral pain, thirst, dysgeusia, or oral cancer may be more present than in healthy controls (Ruospo et al., 2014). However, these symptoms were less studied and therefore not part of this review.

Differences in study design, number of included patients, outcome measurements and country of origin led to differences in outcome measurements. Smaller number of patients lowers the power of the study, while in large cohorts small differences may turn out statistically significant while they are not clinically relevant. Especially the described periodontal parameters differ between the studies, making a good comparison difficult. The quality of dental care varies greatly in the world. Also, costs of oral care and insurance policies differ between countries and they have an effect on the extent to what patients seek dental care. In this review studies from different parts of the world are included and they may partially explain differences between studies.

ESRD patients may be waiting for a kidney transplant. Potential transplant candidates should be free of inflammation before they can receive a transplant, in order to avoid infectious complications when on anti‐inflammatory drugs after transplantation (Sarmento et al., 2020). Since periodontal inflammation and/or dental caries in ESRD patients are common, and low salivary flow may predispose to the rapid progression of these diseases later, a careful examination of the oral cavity and treatment of oral problems before transplantation may be part of the pretransplant procedure.

To conclude, xerostomia and hyposalivation were highly prevalent in ESRD patients. Also, caries and periodontal disease were present. ERSD patients may have more deepened pockets, but not more carious teeth compared to healthy controls.

## CONFLICT OF INTEREST

All authors declare no conflict of interest.

## AUTHOR CONTRIBUTIONS

Conception and design: A. M. G. A. Laheij, W. Rooijers, L. Bidar, L. Haidari, A. Neradova, R. de Vries, and F. R. Rozema. Acquisition of data: A. M. G. A. Laheij, W. Rooijers, L. Bidar, L. Haidari, and R. de Vries. Analysis and interpretation of data: A. M. G. A. Laheij, W. Rooijers, L. Bidar, and L. Haidari. Writing of the manuscript: A. M. G. A. Laheij, W. Rooijers, L. Bidar, L. Haidari, A. Neradova, and R. de Vries, FR. Final approval of the work: A. M. G. A. Laheij, W. Rooijers, L. Bidar, L. Haidari, A. Neradova, R. de Vries, and F. R. Rozema. Agreed to be accountable for the work: A. M. G. A. Laheij, W. Rooijers, L. Bidar, L. Haidari, A. Neradova, R. de Vries, and F. R. Rozema.

## Supporting information


**Table S1** Full search strategies
**Table S2**: Joanna Briggs Institute Critical Appraisal Checklist for Studies Reporting Prevalence Data

## Data Availability

Data sharing is not applicable to this article as no new data were created or analyzed in this study.
