# Supplementary material for: Oral health in patients with end‐stage renal disease: A scoping review
Source: Clin Exp Dent Res. 2021 Aug 29;8(1):54–67. doi: 10.1002/cre2.479 (PMC8874082; doi:10.1002/cre2.479)
Supplement: Supplementary file 1 — Table S1 Full search strategies Table S2: Joanna Briggs Institute Critical Appraisal Checklist for Studies Reporting Prevalence Data [file CRE2-8-54-s001.docx]

***Supplementary Table S1: Full search strategies***

**PubMed Session Results (29 Sep 2020)**

| Search | Query | Items found |
| --- | --- | --- |
| #4 | **#3 NOT ("Animals"[Mesh] NOT "Humans"[Mesh])** | 722 |
| #3 | **#1 AND #2** | 738 |
| #2 | **"Periodontitis"[Mesh] OR "Xerostomia"[Mesh] OR "Dental Caries"[Mesh] OR "xerostomi*"[tiab] OR "hyposalivation*"[tiab] OR "hypo-salivation*"[tiab] OR (("dry"[tiab] OR "dryness"[tiab]) AND ("mouth"[tiab])) OR "periodont*"[tiab] OR "caries"[tiab]** | 161,172 |
| #1 | **"Renal Insufficiency, Chronic"[Mesh] OR "esrd"[tiab] OR "eskd"[tiab] OR (("chronic*"[tiab] OR "end stage*"[tiab] OR "endstage*"[tiab]) AND ("renal*"[tiab] OR "kidney*"[tiab]) AND ("insufficien*"[tiab] OR "failur*"[tiab] OR "disease*"[tiab])) OR "Renal Dialysis"[Mesh] OR "renal dialys*"[tiab] OR "hemodialys*"[tiab] OR "haemodialys*"[tiab] OR "hemodiafiltrat*"[tiab] OR "haemodiafiltrat*"[tiab] OR "hemo-dialys*"[tiab] OR "haemo-dialys*"[tiab] OR "hemo-diafiltrat*"[tiab] OR "haemo-diafiltrat*"[tiab] OR "peritoneal dialys*"[tiab] OR "hemofiltrat*"[tiab] OR "haemofiltrat*"[tiab] OR "hemo-filtrat*"[tiab] OR "haemo-filtrat*"[tiab]** | 272,543 |

**Embase.com Session Results (29 Sep 2020)**

| Search | Query | Items found |
| --- | --- | --- |
| #5 | **#4 NOT ('conference abstract'/it OR 'conference review'/it)** | 1,062 |
| #4 | **#3 NOT ([animals]/lim NOT [humans]/lim)** | 1,226 |
| #3 | **#1 AND #2** | 1,257 |
| #2 | **'periodontitis'/exp OR 'xerostomia'/exp OR 'dental caries'/exp OR 'xerostomi*':ab,ti,kw OR 'hyposalivation*':ab,ti,kw OR 'hypo-salivation*':ab,ti,kw OR (('dry':ab,ti,kw OR 'dryness':ab,ti,kw) AND ('mouth':ab,ti,kw)) OR 'periodont*':ab,ti,kw OR 'caries':ab,ti,kw** | 194,009 |
| #1 | **'chronic kidney failure'/exp OR 'esrd':ab,ti,kw OR 'eskd':ab,ti,kw OR (('chronic*':ab,ti,kw OR 'end stage*':ab,ti,kw OR 'endstage*':ab,ti,kw) AND ('renal*':ab,ti,kw OR 'kidney*':ab,ti,kw) AND ('insufficien*':ab,ti,kw OR 'failur*':ab,ti,kw OR 'disease*':ab,ti,kw)) OR 'hemodialysis'/exp OR 'renal dialys*':ab,ti,kw OR 'hemodialys*':ab,ti,kw OR 'haemodialys*':ab,ti,kw OR 'hemodiafiltrat*':ab,ti,kw OR 'haemodiafiltrat*':ab,ti,kw OR 'hemo-dialys*':ab,ti,kw OR 'haemo-dialys*':ab,ti,kw OR 'hemo-diafiltrat*':ab,ti,kw OR 'haemo-diafiltrat*':ab,ti,kw OR 'peritoneal dialys*':ab,ti,kw OR 'hemofiltrat*':ab,ti,kw OR 'haemofiltrat*':ab,ti,kw OR 'hemo-filtrat*':ab,ti,kw OR 'haemo-filtrat*':ab,ti,kw** | 388,280 |

***Supplementary Table S2****: Joanna Briggs Institute Critical Appraisal Checklist for Studies Reporting Prevalence Data*

|  | Question 1: Was the sample frame appropriate to address the target population? | Question 2: Were study participants sampled in an appropriate way? | Question 3: Was the sample size adequate? | Question 4: Were the study subjects and the setting described in detail? | Question 5: Was the data analysis conducted with sufficient coverage of the identified sample? | Question 6: Were valid methods used for the identification of the condition? | Question 7: Was the condition measured in a standard, reliable way for all participants? | Question 8: Was there appropriate statistical analysis? | Question 9: Was the response rate adequate, and if not, was the low response rate managed appropriately? |
| --- | --- | --- | --- | --- | --- | --- | --- | --- | --- |
| Jung 2020 | Yes | Yes | Yes | Yes | Yes | Yes | Yes | Yes | Not applicable |
| Krizan Smojver 2020 | Yes | Yes | Yes | Yes | Yes | Yes | Yes | Yes | Not applicable |
| Oliveria 2020 | Yes | Yes | Yes | Yes | Yes | Yes | Yes | Yes | Not applicable |
| Pallos 2020 | Yes | Yes | Yes | No | Yes | Yes | Yes | Yes | Not applicable |
| Schütz 2020 | Yes | Yes | Yes | Yes | Yes | Yes | Yes | Yes | Not applicable |
| Misaki 2020 | Yes | Yes | Yes | Yes | Yes | Yes | Yes | Yes | Not applicable |
| Marinoski 2019 | Yes | Yes | Yes | Yes | No | Yes | Yes | Yes | Not applicable |
| Menezes 2019 | Yes | Yes | Yes | Yes | Yes | Yes | Yes | Yes | Not applicable |
| Vaiana-Rojas 2019 | Yes | Yes | Yes | Yes | Yes | Yes | Yes | Yes | Not applicable |
| Bruzda-Zwiech 2018 | Yes | Yes | Yes | Yes | Yes | Yes | Yes | Yes | Not applicable |
| Parente 2018 | Yes | Yes | Yes | Yes | Yes | Yes | Yes | Yes | Not applicable |
| Yue 2018 | Yes | Yes | Yes | Yes | Yes | Yes | Yes | Yes | Not applicable |
| Schmalz 2017 | Yes | Yes | Yes | Yes | No | Yes | Yes | Yes | Not applicable |
| Perozini 2017 | Yes | Yes | Yes | Yes | Yes | Yes | Yes | Yes | Not applicable |
| López-Pintor 2017 | Yes | Yes | Yes | Yes | No | Yes | Yes | Yes | Not applicable |
| Honarmand 2017 | Yes | Yes | Yes | Yes | Yes | Yes | Yes | Yes | Not applicable |
| Palmer 2016 | Yes | Yes | Yes | Yes | Yes | Yes | Yes | Yes | Not applicable |
| Schmalz 2016 | Yes | Yes | Yes | Yes | Yes | Yes | Yes | Yes | Not applicable |
| Naruishi 2016 | Yes | Yes | Yes | Yes | Yes | Yes | Yes | Unclear | Not applicable |
| Bruzda-Zwiech 2014 | Yes | Yes | Yes | Yes | Yes | Yes | Yes | Yes | Not applicable |
| Zhao 2014 | Yes | Yes | Yes | Yes | Yes | Yes | Yes | Yes | Not applicable |
| Gautam 2014 | Yes | Yes | Yes | Yes | Yes | Yes | Unclear | Yes | Not applicable |
| Tadakamadla 2014 | Unclear | Yes | No | Unclear | Yes | Yes | Yes | Yes | Not applicable |
| Jain 2014 | Yes | Yes | Yes | Yes | Yes | Yes | Yes | Yes | Not applicable |
| Ruospo 2014 | Yes | Not applicable | Yes | Unclear | Yes | Yes | Unclear | Yes | Not applicable |
| Swapna 2013 | Yes | Yes | Yes | Yes | Yes | Yes | Yes | Yes | Not applicable |
| Kaushik 2013 | Yes | Yes | Yes | Yes | No | Yes | Yes | Yes | Not applicable |
| Murali 2012 | Yes | Yes | Yes | No | Yes | Yes | Yes | Yes | Not applicable |
| Eltas 2012 | Yes | Yes | Yes | Yes | Yes | Yes | Unclear | Yes | Not applicable |
| Sekiguchi 2012 | Yes | Yes | Yes | Yes | Yes | Yes | Yes | Yes | Not applicable |
| Malekmakan 2011 | Yes | Yes | Yes | Yes | Yes | Yes | Yes | Yes | Not applicable |
| Dirschnabel 2011 | Yes | Yes | Yes | Yes | Yes | Yes | Unclear | Yes | Not applicable |
| Torres 2010 | Yes | No | Yes | Yes | Yes | Yes | Yes | Yes | Not applicable |
| Bayraktar 2008 | Yes | Yes | Yes | Yes | Yes | Yes | Unclear | Yes | Not applicable |
| Sobrado Marinho 2007 | Yes | Yes | No | Yes | Yes | Yes | Yes | Yes | Not applicable |
| Bots 2007 | Unclear | Yes | Yes | Unclear | Yes | Yes | Yes | Yes | Yes |
| De la Rosa García 2006 | Yes | Yes | Yes | Yes | Yes | Yes | Unclear | Yes | Not applicable |
| Bots 2006 | Yes | Yes | Yes | Yes | Yes | Yes | Yes | Yes | Not applicable |
| Chuang 2005 | Yes | Yes | Yes | Yes | Yes | Yes | Yes | Yes | Not applicable |
| Bots 2004 | Yes | Yes | Yes | Yes | Yes | Yes | Yes | Yes | Not applicable |
| Marakoglu 2003 | Yes | Yes | No | Yes | Yes | Yes | Yes | Yes | Not applicable |
| Al-Wahadni & Al-Omari 2003 | Yes | Yes | Yes | Yes | Yes | Yes | Unclear | Yes | Not applicable |
| Gavaldá 1999 | Yes | Yes | Yes | Yes | Yes | Yes | Yes | Yes | Not applicable |
| Kho 1999 | Yes | Yes | Yes | Yes | No | Yes | Yes | Yes | Not applicable |
